# Insulin autoimmune syndrome in a pregnant female: A rare case report: Erratum

**DOI:** 10.1097/MD.0000000000009743

**Published:** 2018-01-26

In the article, “Insulin autoimmune syndrome in a pregnant female: A rare case report”^[1]^, which appeared in Volume 96, Issue 51 of *Medicine*, Dr. Jixiong Xu's name appeared incorrectly as Jix Iong Xu.
